# Simultaneous integrated dose reduction intensity-modulated radiotherapy effectively reduces cardiac toxicity in limited-stage small cell lung cancer

**DOI:** 10.20892/j.issn.2095-3941.2022.0326

**Published:** 2023-06-09

**Authors:** Jing Luo, Jiawei Song, Li Xiao, Jiajia Zhang, Yipeng Cao, Jun Wang, Ping Wang, Lujun Zhao, Ningbo Liu

**Affiliations:** 1 Department of Immunology, Tianjin Medical University Cancer Institute & Hospital, National Clinical Research Center for Cancer, Key Laboratory of Cancer Prevention and Therapy, Tianjin, Tianjin’s Clinical Research Center for Cancer, Key Laboratory of Cancer Immunology and Biotherapy, Tianjin 300060, China; 2 Department of Oncology, Ganyu District People’s Hospital of Lianyungang City, Lianyungang 222100, China; 3 Department of Oncology, Hebei Province Cangzhou Hospital of Integrated Traditional and Western Medicine, Cangzhou 061000, China; 4 Department of Radiation Oncology, Yantai Yuhuangding Hospital, Yantai 264000, China; 5 Department of Radiation Oncology, Tianjin Medical University Cancer Institute & Hospital, National Clinical Research Center for Cancer, Key Laboratory of Cancer Prevention and Therapy, Tianjin, Tianjin’s Clinical Research Center for Cancer, Tianjin 300060, China

**Keywords:** Intensity-modulated radiotherapy (IMRT), small cell lung cancer (SCLC), cardiac irradiation, survival

## Abstract

**Objective:**

To assess the clinical outcomes and toxicities of once daily (QD) simultaneous dose reduction intensity-modulated radiotherapy (SDR-IMRT-QD; SDR-QD) versus conventional QD IMRT (C-QD) and twice daily (BID) IMRT in patients with limited-stage small cell lung cancer (LS-SCLC).

**Methods:**

After propensity score matching (PSM), a retrospective analysis involving 300 patients with LS-SCLC treated using SDR-QD, C-QD, or BID was performed from January 1, 2014 to December 31, 2019. The prescribed irradiation dose in the SDR-QD cohort was 60 Gy/PGTV and 54 Gy/PTV QD. The radiation dose was 60 Gy for both PGTV and PTV QD in the C-QD cohort. The radiation dose was 45 Gy for both PGTV and PTV in the BID cohort. Toxicities, short-term effects, and survival outcomes were recorded. A meta-analysis on the protective effects of pharmaceuticals for cardiac toxicities induced by anti-tumor therapy was performed.

**Results:**

The median overall survival time (MST) in the 3 cohorts were 32.7 months (SDR-QD), 26.3 months (C-QD), and 33.6 months (BID); the differences between groups were statistically significant. Lower toxicities and doses to organs-at-risk (OARs) occurred in the SDR-QD and BID cohorts. Further, the cardiac dose dosimetric parameter Vheart40 was negatively associated with survival (*r* = −0.35, *P* = 0.007). A Vheart40 value of 16.5% was recommended as a cut-off point, which yielded 54.7% sensitivity and 85.7% specificity for predicting negative survival outcomes. The meta-analysis indicated that pharmaceuticals significantly reduced the cardiac toxicities induced by chemotherapy, but not radiotherapy.

**Conclusions:**

SDR-QD was shown to have similar toxicities and survival compared with BID, but fewer toxicities and better survival than C-QD. In addition, cardiac dose exposure was negatively associated with survival. Thus, 16.5% of the cardiac dosimetric parameter Vheart40 is recommended as the cut-off point, and a Vheart40 > 16.5% predicts poor survival.

## Introduction

Small cell lung cancer (SCLC) is a malignant tumor that constitutes > 15% of all lung cancers. Since SCLC is highly malignant, SCLC has become an important cause of cancer-related deaths worldwide^1^. SCLC is categorized into two stages, limited and extensive. According to the tumor-node-metastasis classification method detailed in the 8th American Joint Committee on Cancer Staging Manual, limited stage (LS)-SCLC involves T1-4N1-3M0 lesions that are confined to the ipsilateral hemithorax and can be completely encompassed within a single irradiation portal^2^. Although LS-SCLC is highly sensitive and responsive to anti-cancer therapy, LS-SCLC still has a poor prognosis with a median survival of 16–24 months after curative intent treatment and a 5-year overall survival (OS) rate less than 8%^3–5^. The standard treatment for LS-SCLC is first-line chemotherapy plus thoracic radiation therapy (TRT). The NCCN guidelines have recommended two regimens for the treatment of LS-SCLC [45 Gy twice daily (BID) or 60–70 Gy once daily (QD)]. Although TRT with 45 Gy BID was recommended as standard treatment by the Intergroup 0096 trial and yielded improved survival, this regimen has not been widely adopted. A survey of radiation oncologists reported that 60% of oncologists in the US preferred TRT QD, and 75% reported administering this regimen most often^6^. The prescribing habits in China are quite similar and TRT QD schedules are commonly used for patients with SCLC^7^. Based on the findings of the CONVERT trial and other clinical trials^5,8,9^, a TRT of 45 Gy BID showed a survival equivalent when compared to a TRT of 60–70 Gy QD. In addition, a dose of 45 Gy BID was associated with toxicities^6,10^; however, precise information regarding the toxicities remains unclear, and further exploration of a QD regimen is warranted.

The optimal dose of TRT has not yet been definitively established. Theoretically, higher doses of radiotherapy (RT) for the treatment of tumors improve local tumor control, and several studies have reported a positive association between higher irradiation doses and tumor control rates^11^. The well-known Radiation Therapy Oncology Group (RTOG)-0617 trial demonstrated the optimal RT dose for lung cancer^12,13^, and reported that patients who received a higher dose of radiation (74 Gy) had a significantly shorter survival time compared to patients who received the traditional dose of irradiation (60 Gy). Although the RTOG-0617 trial involved patients with non-small cell lung cancer (NSCLC), the findings are of great significance in radiation therapy for lung cancers in general. Moreover, patients who received higher doses of radiation had a 38% increased risk of death. Although the reasons for this result have not been established, the reduced patient survival might be attributed to radiation-induced adverse effects. Studies that assess optimal radiation patterns to target tumors, while sparing normal peripheral tissues and organs are underway. A previous study reported that different radiation doses are required to control clinical and subclinical lesions, and 50 Gy is an optimal dose for subclinical lesions^14^. Furthermore, immunotherapy has an increasingly important role in comprehensive treatment, and a combination of immune checkpoint inhibitors (ICIs) and TRT requires fewer radiation-related toxicities^15,16^.

SDR-QD is a special pattern of IMRT that involves the delivery of a relatively higher radiation dose to the central lesions and a comparatively lower dose to the subclinical lesions or regions considered to be high risk^17,18^. Thus, the current study was designed to compare SDR-QD as a TRT pattern with BID pattern, which had been confirmed effective for the treatment of patients with LS-SCLC. The feasibility and efficacy of the SDR-QD pattern was assessed, providing evidence-based support for future clinical studies and treatment.

## Materials and methods

After obtaining approval from the Human Investigation Committee of Tianjin Medical University Cancer Institute & Hospital (Approval No. E2019367), a total of 852 patients who received radical TRT at our hospital between January 1, 2014 and December 31, 2019 were recruited as the initial study population. The eligibility criteria included: (I) 18–75 years of age; (II) pathologic evaluation-confirmed SCLC; (III) clinical stage identified by computed tomography (CT), magnetic resonance (MR), ultrasonography, and radionuclide bone scan or positron emission tomography-computed tomography (PET/CT) as the limited stage based on the 6th edition of the American Joint Committee on Cancer; (IV) an inoperable tumor or declined surgery; (V) underwent radical IMRT or IMRT-based chemoradiotherapy; (VI) Karnofsky performance status (KPS) score ≥ 70; and (VII) complete clinical data.

In total, 337 patients underwent SDR-QD, 172 patients underwent C-QD and 105 patients underwent BID were enrolled, respectively. Propensity score matching (PSM) was performed due to the non-randomized selection basis of the study based on the possible confounding variables, such as gender, age, stage of disease, KPS, cigarette smoking, and treatment. After balancing the confounding factors, a total of 300 patients were recruited with 100 patients in each group. All patients in this study signed informed consent.

### Chemotherapy

Patients were administered 2-6 courses of etoposide and cisplatin (EP)/etoposide and carboplatin (EC) chemotherapy: etoposide (100 mg/m^2^, days 1–5) + cisplatin (30 mg/m^2^, days 1–3)/carboplatin [area under the curve (AUC) = 6]. A full dose of chemotherapy drugs was administered if the leukocyte count was within the normal range and a reduced dose if myelosuppression was present.

### Radiotherapy

**Figure 1:**
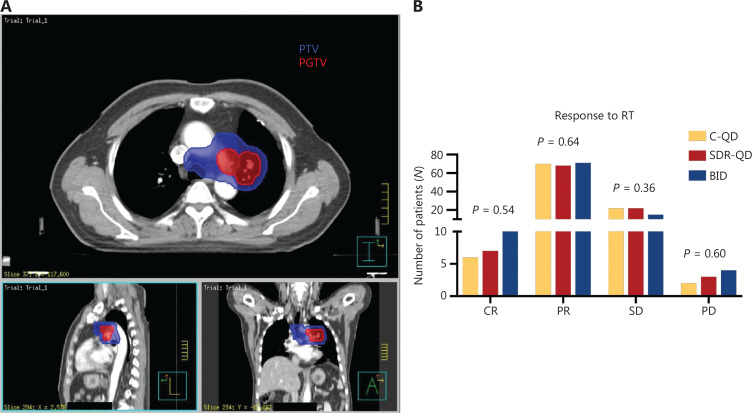
Dose distribution and short-term efficacy. (A) Dose distribution in simultaneous integrated dose reduction intensity-modulated radiotherapy treatment planning. The colored lines represent the following: red, PGTV, receiving 60 Gy; and blue, PTV, receiving 54 Gy. (B) The short-term efficacy was similar between the three groups. PGTV, planning gross tumor volume; PTV, planning tumor volume; C-QD, conventional once daily IMRT; SDR-QD, once-daily simultaneous dose reduction IMRT; BID, twice daily IMRT; IMRT, intensity-modulated radiotherapy.

Radiotherapy commenced with cycles one-to-four of EP/EC chemotherapy; the targets and critical structures were delineated based on the CT images of the Philips Pinnacle^3^ treatment planning system (Philips Medical Systems, Cleveland, Oklahoma, USA) with the assistance of a radiation physicist. Radiation fields were defined according to the changes observed before and after systemic therapy. The gross tumor volume (GTV) was considered as any visible tumor lesions on CT images (lymph nodes identified from CT and/or PET scans were also included). The planning gross tumor volume (PGTV), the clinical target volume (CTV), and the planning target volume (PTV) were defined according to Ming^19^. The prescribed irradiation dose was 60 Gy to PGTV [2.0 Gy/fraction (f) QD] and 54 Gy to the PTV (1.8 Gy/f QD) with 5 fs per week for patients who received SDR-QD. The dose distribution of SDR-QD treatment planning is shown in **Figure 1A**. The prescribed irradiation dose was 60 Gy to PGTV (2.0 Gy/f QD) and 60 Gy to the PTV (2.0 Gy/f QD) with 5 fs per week for patients who received C-QD. The prescribed irradiation dose was 54 Gy to both PGTV and PTV (1.5 Gy/f twice daily) for the BID schedule. The prescription dose covered > 95% of all target volumes. Normal tissue constraints were defined and treatment verification was performed according to local routines as follows: percentage of the total lung volume that received > 5 Gy with the treatment plan Vlung5 ≤ 60%; Vlung20 ≤ 30%; Vlung30 ≤ 20%; percentage of the total heart volume that received > 30 Gy with the treatment plan Vheart30 ≤ 40%; Vheart40 ≤ 30%; and Vheart50 ≤ 60%. For patients who showed favorable clinical and radiographic responses, prophylactic cranial irradiation was recommended with a prescribed irradiation dose of 25 Gy in 10 f, QD^20^.

### Evaluation of treatment outcomes

Patients were evaluated by CT of the brain, chest, and abdomen, and bone imaging in the 1^st^ and 3^rd^ months after chemoRT was completed and every 6 months thereafter. According to the Response Evaluation Criteria in Solid Tumors (version 1.1), short-term efficacy was considered a complete response (CR), partial response (PR), stable disease (SD), or progressive disease (PD). Immeasurable lesions (such as pleural effusions) were not evaluated unless involved in disease progression. All radiation-related cardiac, esophageal, and pulmonary toxicities, as well as myelosuppression, that were clinically diagnosed based on the Common Terminology Criteria for Adverse Events (version 4.0), were evaluated by two oncologists to assess the toxicity type and severity. The OS, progression-free survival (PFS), and locoregional recurrence-free survival (LRFS) were defined according to our previous study^21^. In-field relapse was classified as a recurrence within 95% of the PTV plus a 1 cm margin, whereas out-of-field recurrences were considered intrathoracic lesions outside of the 95% PTV region. Supraclavicular lesions were also included as out-of-field recurrences. Lesions beyond the thorax were considered distant metastases.

### Meta-analysis

The detailed methods and flow chart are provided in **Supplementary Figure S1**.

### Statistical analysis

Data were analyzed using SPSS (version 22.0; SPSS, Inc., Chicago, IL, USA) and R software (version 2.8.0; Institute for Statistics and Mathematics, Vienna, Austria). Kaplan–Meier analyses and log-rank tests were used to measure and compare survival outcomes and treatment failure. Because of the pairwise comparisons, Bonferroni-corrected *P*-values were calculated. According to the Bonferroni formula (α’ = α/m = 0.05/3 = 0.0167) a *P*-value < 0.0167 was considered statistically significant.

Differences between the clinical characteristics of the patients were measured using a chi-squared test. Associations between organs-at-risk (OARs) and survival were analyzed using the Pearson correlation coefficient. PSM analysis with 1:1 matching was performed in every 2 cohorts using a logistic regression estimation algorithm and the nearest neighbor-matching algorithm, with a 0.2 caliper width of the standard deviation. The receiver operating characteristic (ROC) curve was plotted to estimate the cut-off values of the Vheart40 parameter, which optimally demonstrated survival loss, along with the sensitivity, specificity, and AUC with 95% confidence intervals (95% CIs). The survival and ROC curves were drawn using Prism (version 8; GraphPad Software, San Diego, CA, USA). Statistical tests were two-sided, and a *P*-value < 0.05 was considered statistically significant.

## Results

A total of 300 patients were recruited with 100 patients in each group. The median age of the entire population (38% female, 62% male) was 60 years, 96% of patients had a KPS score ≥ 80, 92% had SCLC stage III, and 8% had SCLC stage II. The clinical characteristics of the patients are listed in **Table 1**. The three treatment groups were well balanced after PSM (**Supplementary Table S1**).

**Table 1:** Clinical characteristics of 300 patients with LS-SCLC

		Before matching			After matching			*P*-value
		C-QD	SDR-QD	BID	C-QD	SDR-QD	BID	
Age, years	≥ 60	59	175	49	52	47	47	0.720
	< 60	113	162	56	48	53	53	
Gender	Male	102	181	66	58	64	64	0.490
	Female	70	156	39	42	32	36	
KPS	≥ 80	155	301	97	96	95	97	1.000
	< 80	17	36	8	4	5	3	
Cigarette smoking	Yes	92	207	73	72	66	72	1.000
	No	80	130	32	28	34	28	
PCI	Yes	110	198	65	67	66	62	0.923
	No	62	139	40	33	34	38	
Clinical stage	IIa	8	21	5	4	4	4	0.916
	IIb	6	26	4	3	5	4	
	IIIa	65	123	42	40	38	40	
	IIIb	85	142	47	48	48	46	
	IIIc	8	25	7	5	5	6	
CCRT	Yes	137	229	80	74	75	79	0.530
	No	35	108	25	26	25	21	
Commence CCRT with chemotherapy cycle	1–2	81	182	40	31	33	36	0.620
	3–4	91	155	65	69	67	64	
Gross tumor volume before treatment	< 75.0 cm^3^	108	117	45	43	48	43	0.450
	≥ 75.0 cm^3^	64	220	60	57	52	57	
Adjuvant chemotherapy	Yes	100	216	62	62	54	60	0.550
	No	72	121	43	38	46	40	

C-QD, conventional intensity-modulated radiotherapy (IMRT) once daily IMRT; SDR-QD, simultaneous dose reduction once daily IMRT; BID, twice daily; y, years; KPS, Karnofsky performance status; PCI, prophylactic cranial irradiation; CCRT, concurrent chemoradiothrapy.

### Survival outcomes

**Figure 2:**
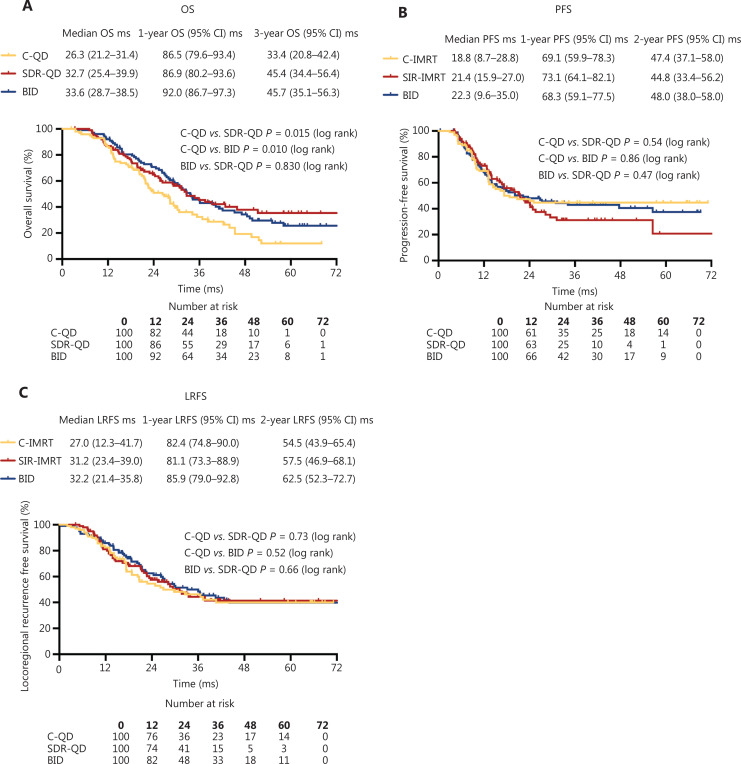
Survival outcomes. (A) Overall survival was longer in the SDR-QD and BID groups than the C-QD group. No statistical differences were observed between the three treatment groups with respect to (B) progression-free survival or (C) locoregional recurrence-free survival. C-QD, conventional once daily IMRT; SDR-QD, once daily simultaneous dose reduction IMRT; BID, twice daily IMRT; IMRT, intensity-modulated radiotherapy; Ms, months.

The response (CR+PR) rate was 75% (75/100) in the SDR-QD group, 76% (76/100) in the C-QD group, and 81% (81/100) after the completion of TRT (**Figure 1B**). No statistically significant differences were observed between the groups. The median overall survival time (MST) of the patients was 30.8 months (95% CI, 27.8–33.9 months), and the median PFS and LRFS were 21.1 months (95% CI, 15.6–25.6 months) and 30.1 months (95% CI, 24.4–35.8 months), respectively. The MST of patients in the SDR-QD, C-QD, and BID groups was 32.7 months (95% CI, 25.4–39.9 months), 26.3 months (95% CI, 22.1–30.5 months), and 33.6 months (95% CI, 28.7–38.5 months), respectively. The differences between the SDR-QD and C-QD groups, and the BID and C-QD groups were statistically significant (*P* = 0.015 and *P* = 0.010, respectively). There was no significant difference between the SDR-QD and BID groups (*P* = 0.83). The median PFS and LRFS of patients in the SDR-QD group were 21.4 months (95% CI, 15.9–27.0 months) and 31.2 months (95% CI, 23.4–39.0 months), respectively. The median PFS and LRFS of patients in the C-QD group were 18.8 months (95% CI, 8.7–28.8 months) and 27.0 months (95% CI, 12.3–41.7 months), respectively. The median PFS and LRFS of patients in the BID group were 22.3 months (95% CI, 9.6–35.5 months) and 33.2 months (95% CI, 21.1–45.3 months), respectively. The survival curves are shown in **Figure 2A–2C**; no significant differences were detected in PFS and LRFS among the three groups.

### Toxicities

**Figure 3:**
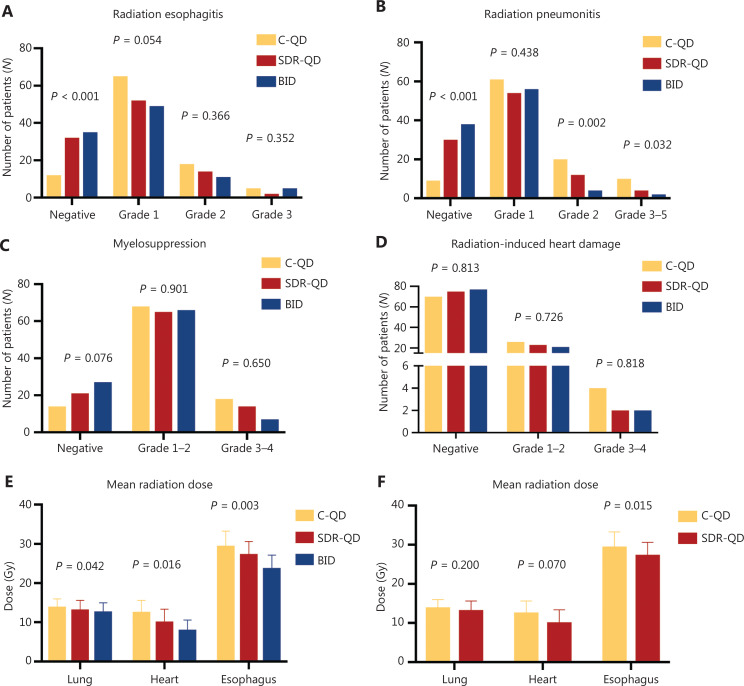
Toxicity profiles and mean irradiation dose rates of different toxicities; specifically, radiation esophagitis (A), radiation pneumonitis (B), myelosuppression (C), and radiation-induced cardiac disease (D) among patients in the C-QD group (yellow), SDR-QD group (red), and BID group (blue). Mean irradiation doses delivered to critical OARs were statistically different from the three groups (E) and two groups (F). C-QD, conventional once daily IMRT; SDR-QD, once daily simultaneous dose reduction IMRT; BID, twice daily IMRT; IMRT, intensity-modulated radiotherapy; OARs, organs-at-risk.

Some of the commonly observed radiation-related toxicities included radiation esophagitis (RE), radiation pneumonitis (RP), radiation-induced cardiac disease (RICD), and myelosuppression. The radiation-related toxicity rates, except RICD, were much lower in the SDR-QD and BID groups than the C-QD group. Differences between the SDR-QD and BID groups were not significant (**Figure 3**). Compared to the C-QD group, the SDR-QD group had markedly reduced RE, RP, and myelosuppression rates (all grades), which was similar to the BID group. RE was less frequent in the SDR-QD and BID groups compared to the C-QD group (32 *vs.* 12 and 35 *vs.* 12, respectively *P* < 0.001). The RE-associated clinical symptoms were more severe among affected patients in the C-QD group than the SDR-QD and BID groups. Similar results were observed with respect to RP and leucopenia. Fewer patients in the SDR-QD and BID groups developed RICD compared to the C-QD group; however, the differences between the groups were not statistically significant.

### Failure

A total of 182 patients had treatment failure in the current study. Fifty-one patients had locoregional failure (18 in-field failure, 20 out-of-field failure, and 13 in-field and out-of-field failures) as the first site of failure and 99 patients developed distant metastasis as the first site of failure. The patterns of failure are outlined in **Table 2**. No differences were observed in the failure patterns between the treatment groups.

**Table 2:** Patterns of treatment failure

Failure		C-QD (*n*)	SDR-QD (*n*)	BID (*n*)	*P*-value
No failure		37	42	39	0.63
Locoregional in-field		6	5	7	0.85
Locoregional in-field + out-of-field		5	3	5	0.83
Locoregional out-of-field		7	5	8	0.81
Distant metastasis only	Bone	7	10	11	0.12
	Brain	14	15	14	0.94
	Liver	7	6	5	0.18
	Others	5	3	2	0.19
Locoregional + Distant		12	11	9	0.26

C-QD, conventional intensity-modulated radiotherapy (IMRT) once daily IMRT; SDR-QD, simultaneous dose reduction once daily IMRT; BID, twice daily.

### Radiation dose delivered to OARs

**Figure 4:**
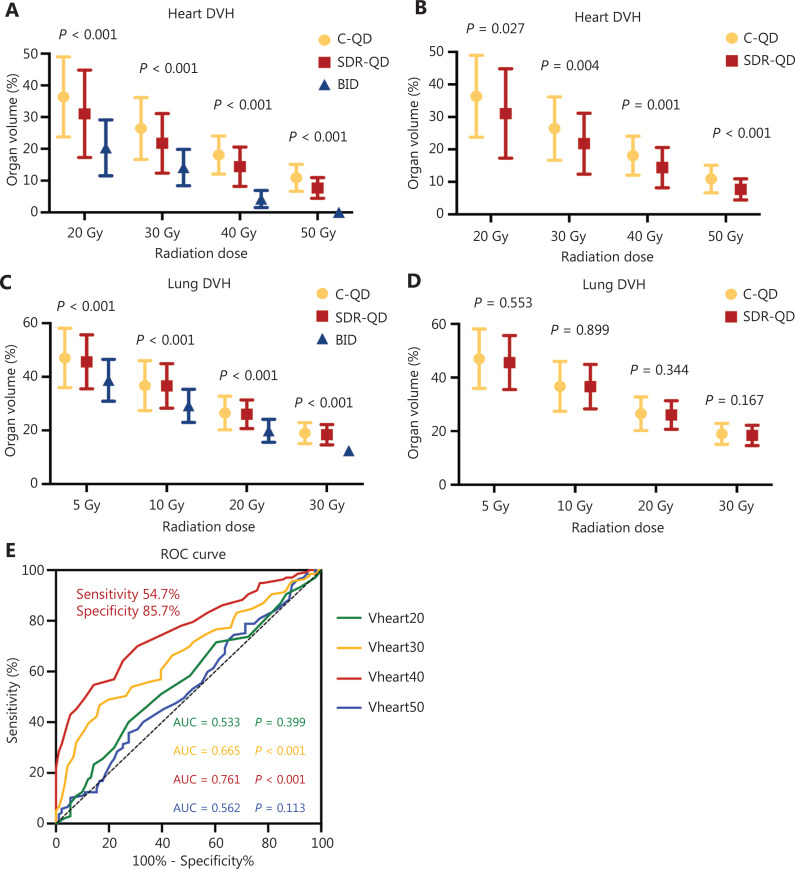
Dosimetric analysis and receiver operating characteristic (ROC) curve. Box plots of DVH data on irradiation dose and OARs volume for the (A) heart and (C) lungs. Differences were demonstrated in the three groups. Compared with the C-QD group, the SDR-QD group also significantly reduced the dose delivered to the heart (B), but not the lungs (D). (E) The ROC curve between heart dosimetric parameters and survival. Vheart40 had the largest AUC (*P* < 0.001). The sensitivity and specificity were 54.7% and 85.7%, respectively. DVH, dose-volume histogram; ROC, receiver operating characteristic; AUC, area under the curve; C-QD, conventional once daily IMRT; SDR-QD, once daily simultaneous dose reduction IMRT; BID, twice daily IMRT; IMRT, intensity-modulated radiotherapy.

Isodose distributions for OARs were evaluated using a dose–volume histogram (DVH). Due to the lower total radiation dose, the treatment plans for the BID group had lower normal tissue-sparing and radiation dosimetric parameters for OARs compared to the SDR-QD and C-QD groups (**Figure 4A and 4B**). Lower OAR parameters were also noted in the SDR-QD group treatment plans compared to the C-QD group; the statistical analysis (**Figure 4C and 4D**) showed the differences to be highly significant, with a *P* < 0.05 for the differences between the doses delivered to the heart and lungs. The SDR-QD group treatment plans had lower percentages of Vheart20–50 compared to the C-QD group (all *P* < 0.05). Interestingly, the dose volume of the lung in the SDR-QD group treatment plans was also lower than the C-QD group treatment plans; however, the difference between the groups was not statistically significant.

The relationship between radiation dosimetric parameters in the OARs and survival rates were also analyzed, as shown in **Table 3**. Correlation analyses showed a statistically weak negative correlation between OS and Vheart40 (*r* = −0.35, *P* = 0.007) and Vheart30 (*r* = −0.128, *P* = 0.053). Indeed, SDR-QD substantially reduced the dose delivered to the heart compared to C-QD, and a lower Vheart40 proportion improved survival. These results are consistent with the survival results, in which patients in the SDR-QD group had longer OS.

**Table 3:** Associations between OARs and survival

	Coefficient	*P*-value
MLD (cGy)	0.037	0.575
MHD (cGy)	−0.052	0.064
Vlung5 (%)	−0.022	0.740
Vlung10 (%)	−0.031	0.641
Vlung20 (%)	−0.060	0.366
Vlung30 (%)	−0.040	0.545
Vheart20 (%)	−0.104	0.116
Vheart30 (%)	−0.268	0.053
Vheart40 (%)	−0.350	0.007
Vheart50 (%)	−0.084	0.205
Esophagus Dmax (cGy)	−0.037	0.576
Vesophagus50 (%)	−0.069	0.324
Cord Dmax (cGy)	−0.044	0.506

MLD, mean lung dose; MHD, mean heart dose.

Patients in whom the OS was shorter than the MST (30.8 months) were considered to have survival loss or poor survival, and patients in whom the OS was longer than the MST were considered to have survival benefit. The ROC curve was plotted, the AUC was calculated, and it was shown that Vheart40 had the largest AUC (0.76; 95% CI, 0.70-0.82) among the 4 heart dosimetric parameters. Therefore, the cut-off value for Vheart40 was evaluated, and 16.5% was identified as the cut-off value that conferred 54.7% sensitivity and 85.7% specificity in predicting survival loss (*P* < 0.001), indicating that high Vheart40 leads to poorer survival outcomes (**Figure 3E**).

### Meta-analysis

Following the flow chart provided in **Supplementary Figure S1**, we finally selected five studies, all of which focused on the protective effect of pharmaceuticals on cardiac toxicities induced by chemotherapy; no studies involving RT were identified. The detailed results are provided in **Supplementary Figure S2**.

## Discussion

Advances in cancer immunotherapies for patients with NSCLC have led to significant improvement in survival^16^, but for patients with LS-SCLC the optimal dose-fractionation schedule of TRT remains debatable. While the Intergroup 0096 trial reported an improvement in survival for BID over QD radiotherapy patterns, traditional standard fractionation (QD) has remained the most common approach over the past decade, and is still very commonly used in the US^22^ and China. Therefore, it is worthwhile to further evaluate the QD schedule. Recently, RT combined with ICIs has led to rapid progress in lung cancer treatment. Immunotherapy has been approved for extensive-stage SCLC and clinical trials are ongoing to evaluate combination RT and immunotherapy in patients with limited-stage SCLC. It seems that immunotherapy combined with twice-daily RT leads to greater toxicity than once-daily RT^23^. In the current study we aimed to deliver adequate irradiation doses to the tumor targets while minimizing the exit dose through critical thoracic OARs. We directly compared the clinical outcomes and toxicities of patients with LS-SCLC after C-QD, SDR-QD, or BID. Although the three groups had similar local and disease control, patients in the SDR-QD and BID groups had longer OS (32.7 and 33.6 months, respectively) than the C-QD group (26.3 months); there were no significant differences between the SDR-QD and BID groups. As reported from the CONVERT trial in 2017^5^, the 45 Gy BID group had a MST of 30 months and a 5-year OS rate of 34%. As reported from the CALGB 30610 trial in 2021^24^, the 45 Gy BID group had a MST of 28.5 months and a 5-year OS rate of 29%, and the 70 Gy QD group had a MST of 30.5 months and a 5-year OS rate of 34%. The 60 Gy/54 Gy QD regimen in the current study had equivalent survival.

The safety of combination therapy is unquestionably important to evaluate because there are mechanistic bases for increased toxicity with combined RT and immunotherapy or chemotherapy^25^; however, the toxicities of RT are expected to be few. Of late, radiation-related lymphopenia was shown to be associated with disease progression and poor survival in lung cancer. Minimizing the dose, especially the lungs and heart doses, reduces lymphopenia and improves survival^15^. Therefore, optimal application and appropriate escalation of irradiation doses, while sparing and minimizing dose delivery to OARs, might be essential to prolong OS and reduce toxicities in patients with SCLC. Because SCLC grows fast and more mediastinal lymph nodes are involved, even in the limited stage, the dose to the mediastinum was reduced in the SDR-QD group, thus protecting important organs, such as the heart and esophagus, from a high dose. The dose to the lungs was not significantly reduced, which could be attributed to the fact that the lungs were the main target that received most of the radiation dose, and the 6 Gy difference in the radiation dose to the PTV was insufficient to generate a statistically significant difference in all lung dosimetric parameters. The OS of patients in the SDR-QD group was similar to the BID group, and longer than patients in the C-QD group, without compromising local or disease control, as well as toxicity. The BID schedule is recommended, but for the QD schedule, a study from the US reported that a radiation dose of 45 Gy is adequate to reduce the incidence of isolated nodal recurrence by > 50%. A dose of 50 Gy eradicated subclinical metastases^13^, thus we believe that a dose of 54 Gy applied to the PTV can effectively control subclinical lesions and reduce the dose delivered to the OARs, which is considered to have a negative relationship with survival. A study from Japan reported that in involved-field radiation therapy, inadvertent irradiation (≥ 40 Gy) might result in a relatively low incidence of elective nodal failure (ENF)^26^. In the current study, elective nodal regions (ENRs) were considered subclinical lesions and included in the target. In addition, when the ENR was applied, only 11.3% (34/300) of patients had locoregional in-field failure. A randomized trial reported by the ASCO in 2020 compared the efficacy of a radiation dose of 45 Gy in 30 f BID with 60 Gy in 40 f BID TRT in patients with LS-SCLC and revealed that patients who received a dose of 60 Gy BID had a statistically significant benefit in 2-year survival and MST compared to patients who received a dose of 45 Gy BID^27^. Although the radiation modalities used in this study may be the most recommended TRT-schedule for LS-SCLC at present, only the 2-year survival of patients was evaluated and long-term survival remains to be assessed. Because our patients were followed for up to 6 years, noticeable results regarding long-term survival were recorded. Therefore, a radiation dose of 60 Gy BID with simultaneous dose reduction TRT of 60 Gy for PGTV and 54 Gy for PTV BID may further improve the OS of patients with LS-SCLC.

Another promising finding of our study was the relationship between heart irradiated volume and survival. In the past few decades, several studies have reported the toxicities and adverse effects of RT; however, compared to RP and RE, the significance of cardiac toxicity has not attracted much attention. RICD is a chronic concern, and is more common among breast, esophageal, and lung cancer patients who undergo chemoradiotherapy^28–30^. We performed a meta-analysis on the protective effects of pharmaceuticals for cardiac toxicities induced by anti-tumor therapy. The results indicated that pharmaceuticals (superoxide dismutase, angiotensin-converting enzyme, and beta-blockers) significantly reduced the cardiac toxicities of patients who receive chemotherapy (**Figure S2**). The MD was 4.26 (1.09-7.03) with a *P*-value of 0.008. Similar studies related to RICD were not identified, suggesting that no pharmaceuticals had protective effects on RICD. It is thus worthwhile to look for new technologies or radiation plans.

It has been reported that the median time-to-diagnosis of RICD is 19 years^31^. Increased survival of patients with cancer and long-term adverse effects, such as cardiac toxicity, have become a focus of research^32^. A study from Japan reported that among patients with esophageal cancer, the potential risk factor for cardiac toxicity after chemoRT was the level of radiation delivered to the heart, and higher exposure was associated with a higher incidence^29^. According to Correa et al.^33^, patients with left breast cancer and prolonged survival were observed to have an increased risk of cardiovascular death after RT. Another study reported that radiation can affect the heart in a dose-dependent manner, and that higher radiation doses (> 40 Gy) significantly increase radiation-induced mortality^31^. Of late, radiation-related lymphopenia has been shown to be associated with disease progression and poor survival in lung cancer. Minimizing the dose, especially the lung and heart doses, can reduce lymphopenia and improve survival^15^; however, the impact of cardiac injury caused by RT on survival remains unclear. The current study attempted to elucidate the ratio of the irradiated heart volume in patients who received SDR-QD and found that this ratio was clearly less than that observed in patients who received C-QD. In addition, the heart dosimetry volume parameter (Vheart40) has a statistically significant negative relationship with OS; however, there were no differences in RICD between the two groups in the current study. This finding could be attributed to patient age because the median patient age in our study was 60 years, and increased age can mask slight cardiac symptoms after chemoRT, which would be defined as a grade I RICD. Another possible reason could be that the patients were followed for up to 72 months. RICD was not given as much attention initially as RE and RP, and some cases were neglected.

Furthermore, the AUC of Vheart40 and survival loss were calculated and a cut-off value of 16.5% was identified, where a value > 16.5% of Vheart40 predicted poorer survival. These results have been further supported by the Lung ART clinical trial^34^ reported by the European Society for Medical Oncology in 2020. According to Lung ART, postoperative conformal radiotherapy did not increase the disease-free survival of patients with NSCLC stage III and mediastinal N2 involvement; however, it did increase early- and late-grade (3–5) cardiopulmonary toxicity. Hence, more patients died due to cardiopulmonary toxicity (16.2% *vs.* 2.0%) or treatment-related toxicities (3.0% *vs.* 0) in the postoperative RT group. A study published in *JAMA Oncology*^35^ also supports our findings. This study focused on adverse cardiac events after RT and showed that the radiation dose delivered to the left anterior descending coronary artery appeared to be an independent risk factor associated with major adverse cardiac events and all-cause mortality in patients with NSCLC, which conversely revealed that the heart irradiation dose was associated with cardiac events and survival. We believe that more attention should be given to the impact of cardiac dose delivery and cardiac toxicity on survival and that evaluation of complete heart dosimetry is important to accurately determine patient outcomes rather than placing emphasis on a single parameter.

### Strengths and limitations

A strength of our study was that it provides the largest cohort of direct comparisons between three radiation modalities (SDR-QD, C-QD, and BID) for the treatment of LS-SCLC. We introduced SDR-QD, a different radiation modality, and verified the effectiveness and safety while also revealing that heart dose was associated with survival and the cut-off value point of Vheart40 was 16.5%, where anything > 16.5% predicts poorer survival. Oncologists should pay more attention to heart exposure during RT, using the clinical cardiac radiation dosimetric parameters as a reference.

Our study had some limitations, including the retrospective design and the lack of a validation cohort. Therefore, prospective validations of the newly identified cardiac exposure constraints are required. In addition, data regarding some clinical symptoms of RICD were neglected, but these details regarding cardiac events will be given more attention in our ongoing prospective study.

## Conclusions

The current study demonstrated that SDR-QD offers several advantages over C-QD since SDR-QD minimizes the underlying dose through critical thoracic OARs. The dose–volume characteristics of the heart, lungs, and esophagus were improved and optimized with SDR-QD; therefore, fewer radiation-related toxicities were observed in the SDR-QD group without compromising local and disease control. In addition, patients in the SDR-QD group had improved OS. We also showed that cardiac dose exposure was negatively associated with survival. A Vheart40 value of 16.5% was recommended as the cut-off point, while a greater value predicted survival loss. Thus, evaluating all heart dosimetry parameters is important to determine patient outcomes, and emphasis should not be placed on a single parameter.
