# Supplementary material for: Simultaneous integrated dose reduction intensity-modulated radiotherapy effectively reduces cardiac toxicity in limited-stage small cell lung cancer
Source: Cancer Biol Med. 2023 Jun 9;20(6):452–64. doi: 10.20892/j.issn.2095-3941.2022.0326 (PMC10291981; doi:10.20892/j.issn.2095-3941.2022.0326)
Supplement: Supplementary file 1 [file cbm-20-452-s001.pdf]

Supplementary materials

Supplementary methods and results of the meta-analysis.

Meta-analysis methods

We systematically searched PubMed, Embase, and the Cochrane Collaboration Central Register of Controlled Trials from inception to 30 May 2022. Keywords and medical subject headings (MeSH) included "chemotherapy," "radiotherapy," and "cardiotoxicity" for the search strategies. Studies were selected according to the following clinical trial criteria: (1) controlled randomization; (2) pharmaceuticals were used as an experimental intervention for cardiac toxicities, regardless of treatment duration and dosage; (3) patients receiving anti-tumor therapy, including chemotherapy or radiotherapy; and (4) reported clinical data before and after therapy. Trials that enrolled patients of any age, gender, or ethnic origin were eligible. The mean difference (MD, which was used for comparison of the decrease in left ventricular ejection fractions (LVEF) in experimental and control groups was considered as end indices. Results are presented with the corresponding 95% confidence interval (CI). Heterogeneity across each study was tested by Cochrane's Q statistic and the I^2 statistic was applied to quantify the heterogeneity ($I^2 = 86\%$ represents substantial heterogeneity); the random-effect model was used in this study.

Results of the meta-analysis

Following the flow chart provided in **Supplementary Figure S1**, we finally selected five studies, all of which focused on the protective effect of pharmaceuticals on cardiac toxicities induced by chemotherapy; no studies involving radiotherapy were identified. Details of the five studies are provided in **Supplementary Figure S2A**. The general characteristics of the individual trials are shown in **Figure S2B**. Considering the heterogeneity of different interventions and patient characteristics, a random-effect model analysis was performed, the results of which indicated that pharmaceuticals (superoxide dismutase, angiotensin-converting enzyme, and beta-blockers) significantly reduced the decrease in LVEF among patients who received chemotherapy; the MD was 4.26 (1.09–7.03) with a *P*-value of 0.008. The meta-analysis demonstrated that cardiac toxicities induced by chemotherapy could be protected by pharmaceuticals, but no pharmaceuticals were observed to release RICD; thus, it is worthwhile to explore new radiation technologies and treatment plans.

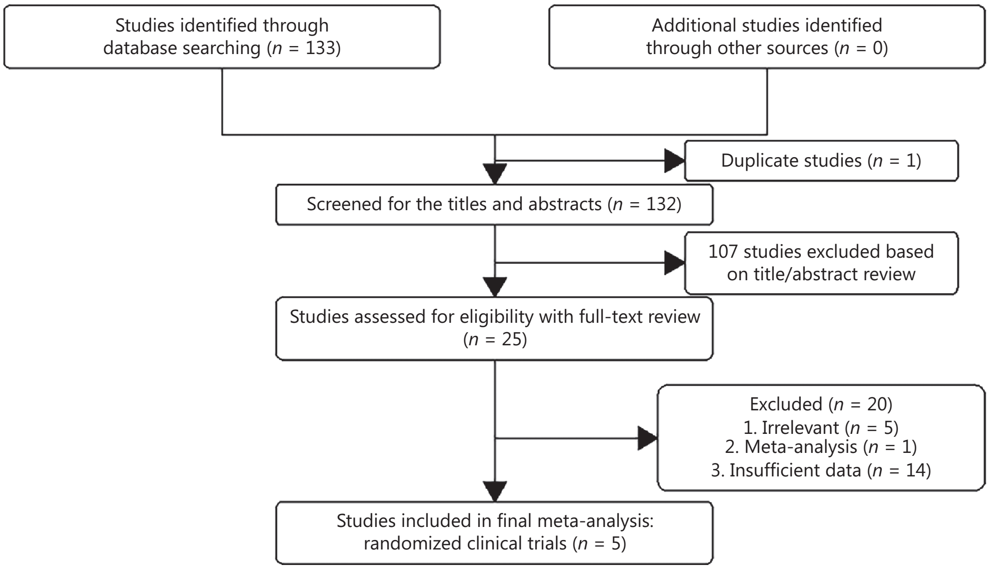

Figure S1 Flow chart of study selection. Protective effect of pharmaceuticals on cardiac toxicities induced by anti-tumor therapy. Five studies were selected; related studies of radiation-reduced cardiac toxicities were not observed.

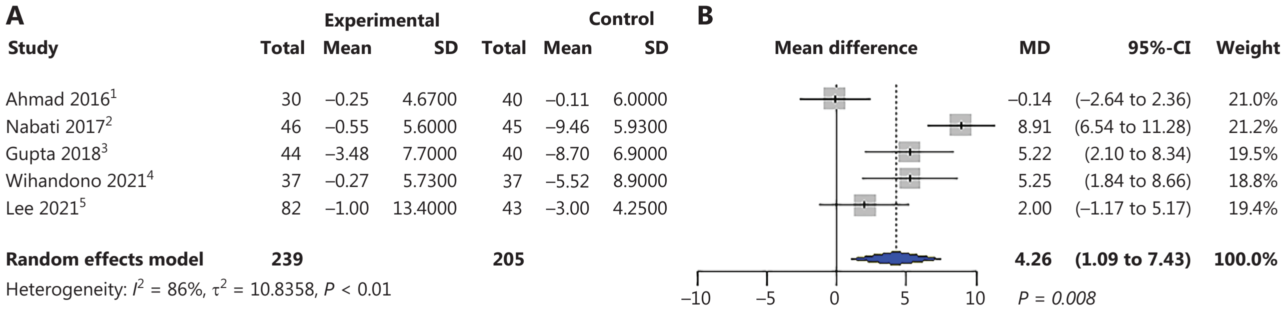

Figure S2 General characteristics of the individual trials (A) and the forest plot showing the MD comparison (B).

Table S1 Clinical characteristics of 300 patients in the SDR-QD, C-QD, and BID groups before and after propensity score matching

		Before matching			After matching			*P*-value
		C-QD	SDR-QD	BID	C-QD	SDR-QD	BID	
Age, y	≥ 60	59	175	49	52	47	47	0.720
	< 60	113	162	56	48	53	53	
Gender	Male	102	181	66	58	64	64	0.490
	Female	70	156	39	42	32	36	
KPS	≥ 80	155	301	97	96	95	97	1.000
	< 80	17	36	8	4	5	3	
Cigarette smoking	Yes	92	207	73	72	66	72	1.000
	No	80	130	32	28	34	28	
PCI	Yes	110	198	65	67	66	62	0.923
	No	62	139	40	33	34	38	
Clinical stage	IIa	8	21	5	4	4	4	0.916
	IIb	6	26	4	3	5	4	
	IIIa	65	123	42	40	38	40	
	IIIb	85	142	47	48	48	46	
	IIIc	8	25	7	5	5	6	
CCRT	Yes	137	229	80	74	75	79	0.530
	No	35	108	25	26	25	21	
Commence CCRT with chemotherapy cycle	1–2	81	182	40	31	33	36	0.620
	3–4	91	155	65	69	67	64	
Gross tumor volume before treatment	< 75.0 cm^3	108	117	45	43	48	43	0.450
	≥ 75.0 cm^3	64	220	60	57	52	57	
Adjuvant chemotherapy	Yes	100	216	62	62	54	60	0.550
	No	72	121	43	38	46	40	

y, years; KPS, Karnofsky performance status; PCI, prophylactic cranial irradiation; CCRT, concurrent chemoradiothrapy; MST, median overall survival time; C-QD, conventional once daily intensity-modulated radiotherapy; SDR-QD, simultaneous dose reduction intensity-modulated radiotherapy; BID, twice daily intensity-modulated radiotherapy.

References

1. Beheshti AT, Toroghi HM, Hosseini G, Zarifian A, Shandiz FH, Fazlinezhad A. Carvedilol Administration can prevent doxorubicin-induced cardiotoxicity: a double-blind randomized trial. Cardiology. 2016; 134: 47-53.
2. Nabati M, Janbabai G, Baghyari S, Esmaili K, Yazdani J. Cardioprotective Effects of Carvedilol in inhibiting doxorubicin-induced cardiotoxicity. J Cardiovasc Pharmacol. 2017; 69: 279-85.
3. Gupta V, Singh SK, Agrawal V, Singh TB. Role of ACE inhibitors in anthracycline-induced cardiotoxicity: a randomized, double-blind, placebo-controlled trial. Pediatr Blood Cancer. 2018; 65: e27308.
4. Wihandono A, Azhar Y, Abdurahman M, Hidayat S. The role of lisinopril and bisoprolol to prevent anthracycline induced cardiotoxicity in locally advanced breast cancer patients. Asian Pac J Cancer Prev. 2021; 22: 2847-53.
5. Lee M, Chung W-B, Lee J-E, Park C-S, Park W-C, Song B-J, et al. Candesartan and carvedilol for primary prevention of subclinical cardiotoxicity in breast cancer patients without a cardiovascular risk treated with doxorubicin. Cancer Med. 2021; 10: 3964-73.
